# Measuring Food Insecurity in India: A Systematic Review of the Current Evidence

**DOI:** 10.1007/s13668-023-00470-3

**Published:** 2023-04-06

**Authors:** Fiona H. McKay, Alice Sims, Paige van der Pligt

**Affiliations:** 1 School of Health and Social Development, Faculty of Health, Deakin University, Victoria, Australia; 2 Institute for Health Transformation, Faculty of Health, Deakin University, Victoria, Australia; 3 Institute for Physical Activity and Nutrition (IPAN), School of Exercise and Nutrition Sciences, Deakin University, Geelong, Australia

**Keywords:** India, Food insecurity, Measurement, Nutrition, Health, Review

## Abstract

**Purpose of Review:**

India is home to an estimated 200 million malnourished people, suggesting widespread food insecurity. However, variations in the methods used for determining food insecurity status mean there is uncertainty in the data and severity of food insecurity across the country. This systematic review investigated the peer-reviewed literature examining food insecurity in India to identify both the breadth of research being conducted as well as the instruments used and the populations under study.

**Recent Findings:**

Nine databases were searched in March 2020. After excluding articles that did not meet the inclusion criteria, 53 articles were reviewed. The most common tool for measuring food insecurity was the Household Food Insecurity Access Scale (HFIAS), followed by the Household Food Security Survey Module (HFSSM), and the Food Insecurity Experience Scale (FIES). Reported food insecurity ranged from 8.7 to 99% depending on the measurement tool and population under investigation. This study found variations in methods for the assessment of food insecurity in India and the reliance on cross-sectional studies.

**Summary:**

Based on the findings of this review and the size and diversity of the Indian population, there is an opportunity for the development and implementation of an Indian-specific food security measure to allow researchers to collect better data on food insecurity. Considering India’s widespread malnutrition and high prevalence of food insecurity, the development of such a tool will go part of way in addressing nutrition-related public health in India.

**Supplementary Information:**

The online version contains supplementary material available at 10.1007/s13668-023-00470-3.

## Introduction

Food insecurity has been identified as a “pressing public health concern” in India [1•]. At the household level, food security exists when all members, at all times, have access to enough food for an active, healthy life [2••]. Individuals who are food secure do not live with hunger or fear starvation. Across urban settings, the prevalence of food insecurity has been found to range from 51 to 77%, yet over 70% of India’s population resides rurally, where data concerning food insecurity is limited [3].

The concept of food security consists of six main dimensions: availability, access, utilization, stability, agency, and sustainability. The first three dimensions are interlinked and hierarchical. Food availability is concerned with ensuring that sufficient quantities of food of appropriate quality are supplied through domestic production or imports (including food aid). Access to food is necessary but not sufficient for access. Access is concerned with ensuring adequate resources, or entitlements, are available for the acquisition of appropriate foods for a nutritious diet. Access is necessary but not sufficient for utilization. Utilization is concerned with the ability of an individual to access an adequate diet, clean water, sanitation, and health care to reach a state of nutritional well-being. The three other concepts have become increasingly accepted as important, as risks such as climatic fluctuations, conflict, job loss, and epidemic disease can disrupt any one of the first three factors. Stability refers to the constancy of the first three dimensions. Agency is recognized as the capacity of individuals or groups to make their own food decisions, including about what they eat, what and how they produce food, and how that food is distributed within food systems and governance. Finally, sustainability refers to the long-term ability of food systems to provide food security and nutrition in a way that does not compromise the economic, social, and environmental bases that generate food security and nutrition for future generations [4••].

Two hundred million people living in India are estimated to be malnourished [5•]. Poverty, a lack of clean drinking water, and poor sanitation have been identified as common factors contributing to malnutrition in India [1•]. Yet to date, despite high rates of malnutrition pointing toward widespread food insecurity [6], the link between food insecurity and malnutrition in India has seldom been explored. Of the limited data available, associations have been found between household food insecurity and child stunting, wasting, and being underweight [7], highlighting the urgency of food insecurity as a public health priority.

Considering the high rates of child stunting, wasting, and overall malnutrition in India, exploring past and emerging research which has both assessed and addressed food insecurity is a crucial step in better understanding nutrition-related health at the population level. Currently, to the best of our knowledge, there is no published systematic review which has explored household food insecurity in India. To understand the factors that contribute to food insecurity at the household level, the related health and nutrition outcomes, and to conceptualize potential strategies which target food insecurity in India, a systematic review of published research undertaken to date which has focused on food insecurity in India is urgently needed. This review seeks to (1) systematically investigate the peer-reviewed literature that purports to investigate food insecurity in India, (2) identify the breadth of research being conducted in India, including the instruments used and the populations under study, and (3) provide an overview of the severity of food insecurity in India as presented by these studies.

## Method

**Fig. 1:**
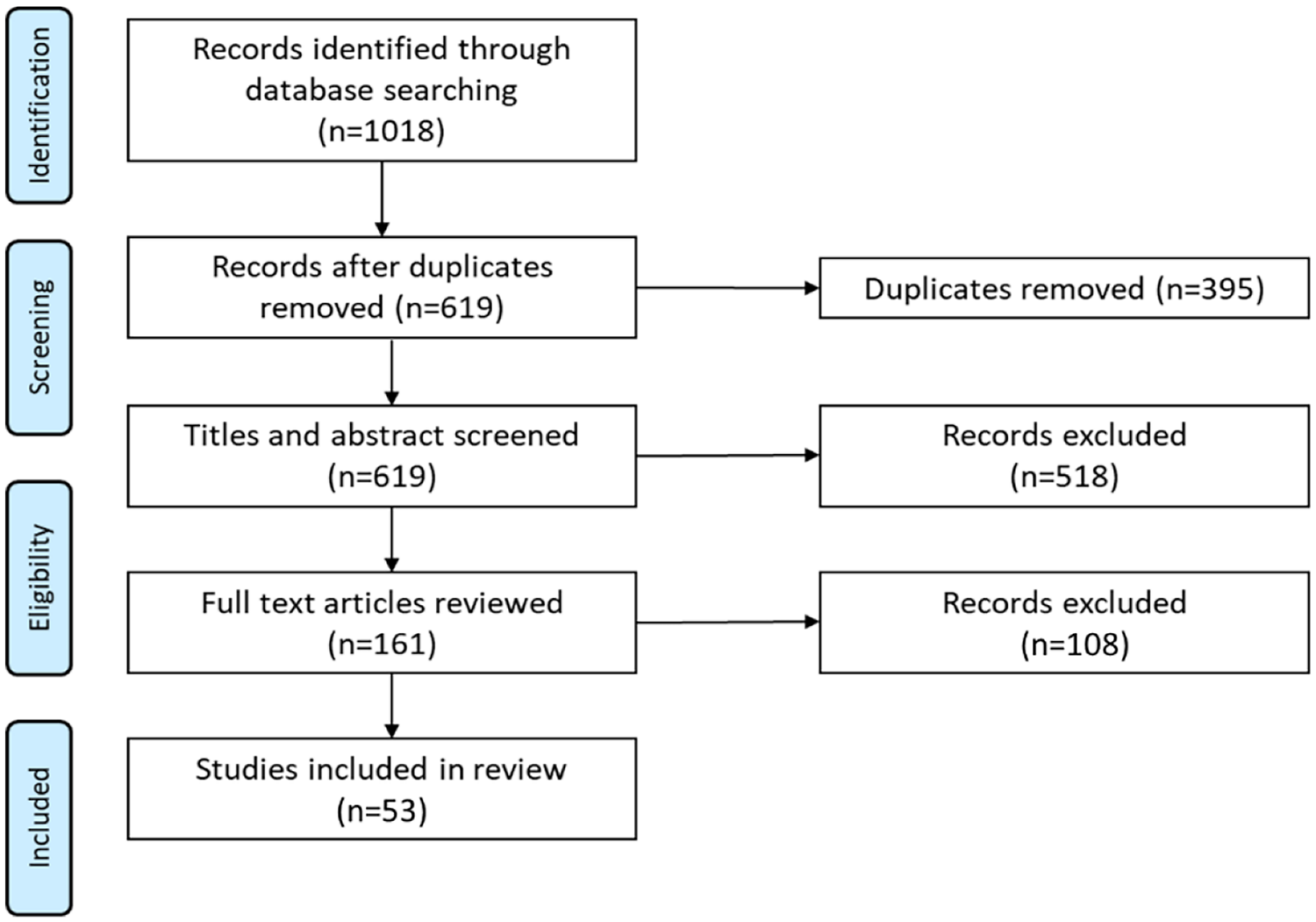
Flow chart of articles meeting search criteria, number of articles excluded, and final number of articles meeting inclusion criteria for review

A systematic search was undertaken to identify all food security research conducted at the household level in India. The search was conducted in March 2020. Key search terms were based on the FAO [8] definition of food security: “food access*,” OR “food afford*,” OR “food insecur*,” OR “food poverty*,” OR “food secur*,” OR “food suppl*,” OR “food sufficien*,” OR “food insufficien*,” OR “hung*” AND “household*” OR “house*” AND “India.” Searched databases included Academic Search Complete, CINAHL Complete, Global Health, MEDLINE, Embase, SCOPUS, ProQuest, PsychInfo, and Web of Science. To gain a full collection of articles that reported on research investigating household food security in India, no limits were placed on publication dates. Only peer-reviewed articles published in English were considered; unpublished articles, books, theses, dissertations, and non-peer-reviewed articles were excluded. This review adheres to the PRISMA statement [9, 10], see Fig. 1 for a flowchart describing the process of screened included and excluded articles.

Two authors (FHM and AS) and a research assistant reviewed all articles to identify relevant studies. Articles underwent a three-step review process (see Fig. 1). All articles were downloaded into EndNote X7, duplicates were identified and removed, and the article titles, journal titles, year, and author names were then exported to Microsoft Excel 365 to facilitate reviewing. Articles were first screened by title and abstract based on the inclusion and exclusion criteria described above by two authors independently. Any article that clearly did not meet the inclusion criteria was removed at this stage, any that did, or possibly could meet the inclusion criteria on further inspection, were retained. The full text of the remaining articles was obtained, and at least two authors (FHM and AS) or a research assistant independently read all 161 articles that remained at this stage to determine if the article met the inclusion criteria. Any articles at this stage that clearly did not meet the inclusion criteria were removed. Any disagreements on those that were retained were discussed and settled by consensus between the authors.

Articles that discussed food insecurity in general but collected no new data (for example, Gopalan [11] and Gustafson [12]) were excluded, as were previously conducted reviews in the region (for example, del Ninno, Dorosh [13], Harris-Fry, Shrestha [14]). As this review was primarily interested in studies that purported to measure food insecurity in India, studies that discussed food insecurity, either as the standard measured construct or as a construct created by the authors but termed food insecurity, were included. While there are many non-government organizations and inter-government organizations that work to measure food or nutritional insecurity, the construct of “hunger,” the associated conditions of malnutrition (either with overweight or obesity) or conditions that might indicate malnutrition (including anemia or under-5 mortality), these reports generally do not include a complete description of the method used to collect data if data were collected at the household level and often use the sale or production of crops as a proxy; as such, these reports have been excluded from this review.

Data were extracted from each article by the three authors. Data were extracted into a Microsoft Excel 365 spreadsheet that allowed for the capture of specific information across all included articles. Data extracted at this stage included the following: location; population group; findings; measured food security (Y/N); method for determining food insecurity; and prevalence of food insecurity.

## Results

The search identified 1018 articles, of which 395 were duplicates. The titles and abstracts of the remaining 616 articles were read, with 518 articles excluded as they did not refer, either directly or indirectly, to food insecurity research in India, leaving 161 articles for further investigation. The full text of the 161 articles was reviewed; 108 articles were excluded as they did not meet the inclusion criteria. The remaining 53 articles were included in this review.

Most articles (*n* = 48, 90%) were cross-sectional studies; three were longitudinal, with data covering 27 years [15], 11 years [16], and 4 years [17], and one was a randomized controlled trial [18]. Eight studies employed a mixed methods approach, seven were qualitative, and the remaining 38 were quantitative studies. Participant numbers ranged in size from the smallest study with 10 participants [19] to population-level studies with over 100,000 participants [15, 20]. See the supplementary material for an overview of the studies included.

**Fig. 2:**
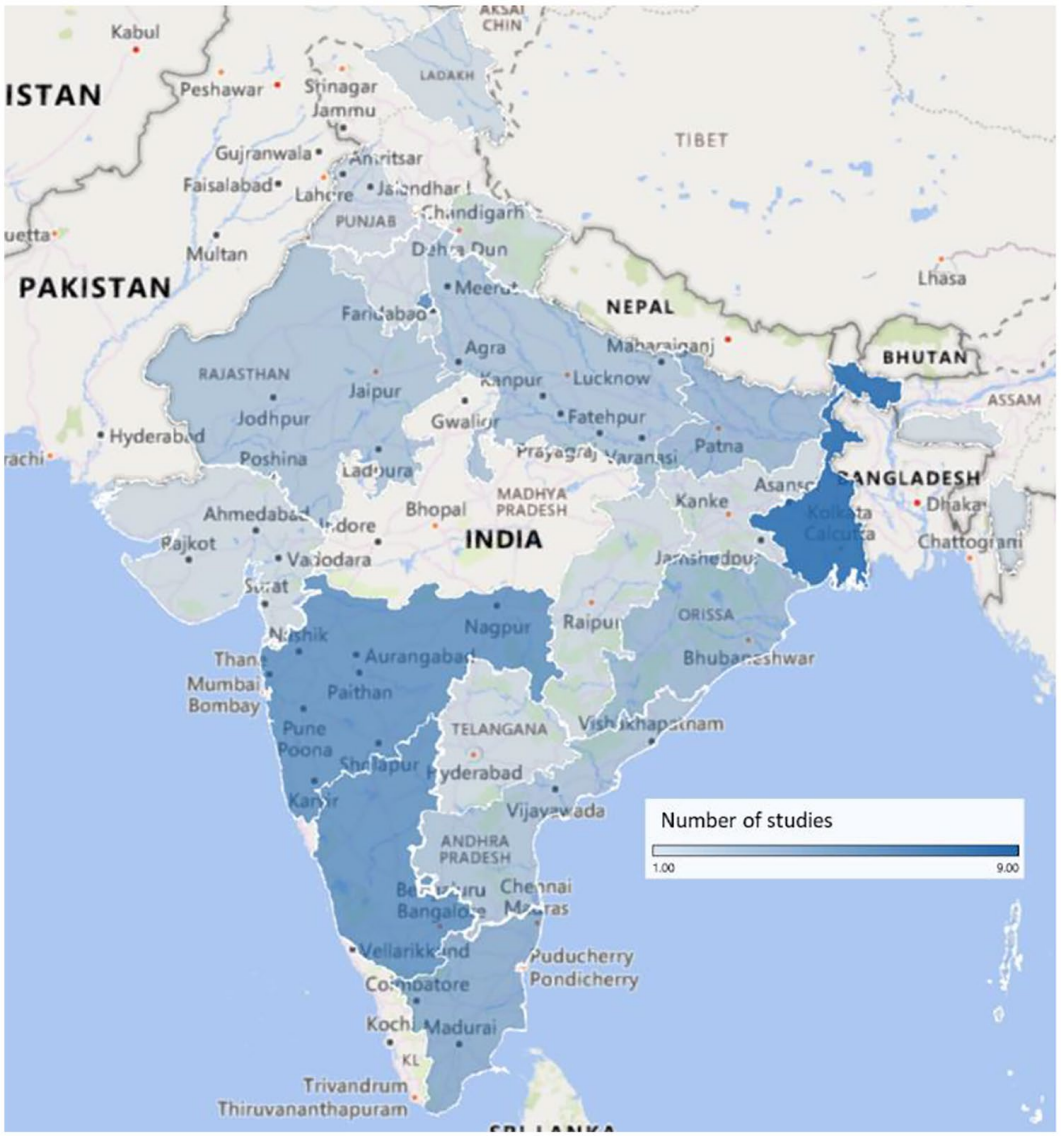
Distribution of studies exploring food insecurity in India

Most food insecurity research was conducted in the state of West Bengal, where 9 studies were conducted, followed by 6 studies each in Maharashtra and the union territory of Delhi (see Fig. 2). India consists of 28 states and 8 union territories; this review found research from 17 states and five union territories, as well as four nationwide studies showing good coverage across the country.

## Measuring Food Insecurity

All studies included in this review purported to measure food insecurity directly, with the main aim of the majority (*n* = 45, 85%) of articles to determine the prevalence of food insecurity. These articles employed a range of measurement tools to achieve this aim. The most common way to measure food insecurity was via the Household Food Insecurity Access Scale (HFIAS) which was employed in 17 studies. The second most common method employed to measure food insecurity was via the Household Food Security Survey Module (HFSSM), employed in 13 studies. Other measures of food insecurity include the Food Insecurity Experience Scale (FIES), used in three studies, the Comprehensive Nutrition Survey in Maharashtra used in two studies, and the Radimer/Cornell used in one study. The remaining 17 studies used a proxy measure, either one devised by the authors or by using data from the India National Sample Survey (NSS). See Table 1 for an overview of these measurement tools.

**Table 1:** Food insecurity measurement tools

**Measure of food insecurity**	**Dimension of food insecurity measured**	**Items**	**Brief description of tool**	**Validated**
Household Food Insecurity Access Scale (HFIAS)	Access	9	The method is based on the idea that the experience of food insecurity (access) causes predictable reactions and responses that can be captured and quantified through a survey and summarized in a scale	Yes [69]
Household Food Security Survey Module (HFSSM)	Access	18	The set of food security questions takes into consideration the overall food insecurity experience and categorizes this phenomenon by its severity	Yes [70, 71]
Food Insecurity Experience Scale (FIES)	Access	8	FIES is a food insecurity severity experience matrix that relies on immediate responses of respondents to questions about their access to adequate food	Yes [72]
National Sample Survey (NSS)	Access (only household expenditure)	Varies	The National Sample Survey (NSS) is a nationally representative survey of the all-India non-institutionalized population	No
Comprehensive Nutrition Survey in Maharashtra (CNNS/M)	Availability (only dietary diversity)	9	The CNNS is a state-specific nutrition survey with a focus on infants and children under two and their mothers	No
Radimer/Cornell	Availability	10	Radimer/Cornell measures of hunger and food insecurity based on interviews	Yes [36]

The prevalence of food insecurity in these studies ranged from 8.7 to 99%; 13 studies stated that they measured food insecurity but did not report food insecurity results. The most common way for food insecurity to be measured in India was through employing Household Food Insecurity Access Scale (HFIAS). This experiential scale was designed to be used cross-culturally and consists of nine questions, with frequency questions asked if participants experience the condition. Responses to these questions are scored so that “never” receives a score of 0, “rarely” is scored 1, “sometimes” is scored 2, and “often” is scored 3, so that when summed, the lowest possible score is 0 and the highest is 27. A higher score represents greater food insecurity, with continuous scores typically divided into four categories, representing food-secure and mildly, moderately, and severely food-insecure households according to the scheme recommended by the HFIAS Indicator Guide [21]. The scale is based on a household’s experience of problems regarding access to food and represents three aspects of food insecurity found to be universal across cultures [22–24]. This scale measures feelings of uncertainty or anxiety about household food supplies, perceptions that household food is of insufficient quality, and insufficient food intake [21]. The questions asked in the HFIAS allow households to assign a score along a continuum of severity, from food secure to food insecure. Food insecurity measured via the HFIAS ranged from 77.2% in a population of 250 women who resided in an urban area in South Delhi [25] to 8.7% in Indian children [26].

The second most common measurement tool identified in this search is the US Household Food Security Survey Module (HFSSM). This tool was developed to measure whether households have enough food or money to meet basic food needs and what their behavioral and subjective responses to that condition were [27]. The HFSSM module consists of a set of 18 items, 8 of which are specific to households with children. It captures four types of household food insecurity experiences: (1) uncertainty and worry, (2) inadequate food quality, and insufficient food quantity for (3) adults and (4) children [28]. It is available in an 18-item and 6-item forms and allows households to be assigned a category of food insecurity: high food security, marginal food insecurity, low food insecurity, and very low food insecurity. In accordance with the method proposed by Coleman-Jensen et al. [29], food security scores are combined to create one measure for the level of food security for a household. Food security status is determined by the number of food-insecure conditions and behaviors that the household reports. Households are classified as food secure if they report fewer than two food-insecure conditions. They are classified as food insecure if they report three or more food-insecure conditions, or two or more food-insecure conditions if they have children. Food-insecure households are further classified as having either low food security if they report between three and five food-insecure conditions (or three and seven if they have children), or very low food security if they have six or more food-insecurity conditions (eight if they have children). Studies that employed the HFSSM reported food insecurity ranging from 15.4 [30–32] to over 80% of study participants [33]. The HFSSM is a commonly used measure of food insecurity and can be used in several valid forms. Studies included in this review used the 4-, 6-, and 18-item versions of the HFSSM.

The Food Insecurity Experience Scale (FIES) module was used by three studies included in this review. The FIES questions refer to the experiences of the individual or household. This scale was created by the Food and Agriculture Organization of the United Nations (FAO) and has been tested for use globally [28]. The questions focus on self-reported food-related behaviors and experiences associated with increasing difficulties in accessing food due to resource constraints. The FIES allows for the calibration of other measures, including the HFIAS and the HSSM with the FIES against a standard reference scale allowing for comparability of the estimated prevalence rates of food insecurity [34], as well as a raw score that can be used by authors as a way to create discrete categories of food insecurity severity [35]. The three studies that employed the FIES all reported food insecurity within a range of 66–77%, despite different population groups, locations, and sample sizes.

One study employed the Radimer/Cornell scale, a widely used and validated scale [36]. The scale includes ten items that relate to food anxiety and the quantity and quality of food available. The instrument allows for the categorization of households into four categories of food insecurity: food security, household food insecurity, individual food insecurity, and child hunger.

The Comprehensive National Nutrition Survey (CNNS) was used in two studies. It is a state-specific (Maharashtra) nutrition survey with a focus on infants and children under two and their mothers. The CNSM measured household food security using nine questions [37]. The questions capture experiences of uncertainty or anxiety over food, insufficient quality, insufficient quantity, and reductions in food intake [38]. Households are categorized as food secure, mildly food insecure, moderately food insecure, or severely food insecure.

The National Sample Survey (NSS) organization conducts nationwide household consumer expenditure surveys at regular intervals in “rounds,” typically 1 year. These surveys are conducted through interviews with a representative sample of households [20]. This survey includes only one question about household daily access to food [39], and while it does provide a method for estimating food insecurity in India, it assumes that financial access equates to physical access to available food; as such, this survey is unlikely to be able to comprehensively capture the intensity of household food insecurity in India [40]. Four studies employed the NSS. Given that these studies did not specifically collect food insecurity data, the use of the NSS has been considered a proxy indicator here as it generally reflects the measurement of food availability or acquisition rather than food insecurity per se.

Other proxy measures were commonly used. The variety of proxy measures included information on calorie intake, purchasing power, the quantity of food consumed, and agricultural productivity. These proxy measures provide only a partial, usually indirect, measure of food insecurity [41]. There are also challenges with these measures, as the relationship between food and caloric quantity and household food security is unpredictable [42]. For example, in a study of households in Gujarat, Sujoy [43] found that around 85% of households are food insecure at some point in a typical year. This study employed a range of measures to explore the experiences of hunger and food insecurity and the strategies employed by these population groups to mitigate hunger. Exploring the food insecurity experiences of farmers in Bihar, Sajjad and Nasreen [44] found that 75% of households had very low food security. While not using a standard measure, Sajjad and Nasreen [44] interviewed households alongside interviews with government officials, food production, food costs, and food acquisition to form an index of food security that could be applied at the household level. A study by George and Daga [45] using calorie consumption as a proxy for food security identified 57% of participants were food insecure, with the suggestion that income and family size play a role in food security among children. Of the 17 studies that employed a proxy measure of food insecurity, 10 provided no indication of the level of food security in their results.

## Population Groups Under Investigation

Studies identified in this review included a variety of population groups. Most studies (*n* = 30) focused on food insecurity at the household level; half of these studies employed one of the standard food insecurity measurement tools, while the other half relied on proxy measures.

Fourteen studies focused specifically on young children, and one on teenagers. These studies used a variety of methods to determine food insecurity among this population, with rates of food insecurity shown to range from 8.7 [26] to 80.3% [33]; within this range, most studies reported that food insecurity among children was in the range of 40 to 60%. Interestingly, while the study conducted by Humphries [26] reported lower levels of child food insecurity (8.7%) than the other studies included in this review, other findings of this study were consistent with other research reviewed. Across all studies that explored food insecurity among children and teenagers, findings suggest problematic infant and young child feeding practices, caregiving, and hygiene practices, with many studies reporting impaired growth in children and teenagers due to these practices.

Seven studies focused specifically on the experiences of women or used the experiences of women as an indicator of food insecurity in their households. All of these studies employed one of the standard measures of food insecurity, with food insecurity in these studies ranging from 32 [3] to 77.9% [46]. These studies identified a range of health outcomes related to food insecurity and hunger. For example, in a study of mothers of children under the age of 5, Das and Krishna [47] found that two-thirds of households were food insecure and that younger mothers were more likely to be food insecure, with the children of these mothers more likely to be underweight and stunted. Among mothers in a study by Chyne et al. [48], those who had low literacy levels, low income, and large family size were more likely to be food insecure, with many of the children of these mothers being vitamin A deficient, anemic, stunted, and/or wasted. This is consistent with the work of Chatterjee et al. [49] who found that food insecurity among women was associated with low income and a range of socioeconomic measures including education, employment, and relationship status.

Thirteen studies were conducted in slums. Four of these studies were conducted in slums in Delhi, finding that food insecurity among slum populations ranges between 12% among children aged 1–2 years [50] and 77% in households more broadly [25]. Three studies were located in slums in Kolkata, all conducted by Maitra and colleagues [30–32]. These studies found food insecurity to be 15.4%, finding that low income, household composition, and education are all predictors of household food insecurity. The remaining studies were conducted in slums in Jaipur [51], Mumbai [49], Varanasi [52], Vellore [53], and West Bengal [33, 54]. Slums are an important setting for an exploration of food insecurity, especially in India, where 25% of the urban population resides in slums or slum-like settings. People living in slums have been found to have poorer quality of life, are generally lower income, and have lower educational attainment than non-slum-dwelling populations—all factors that are known to contribute to food insecurity [49].

Five studies explored food insecurity among people with an underlying health condition. Four of these explored food insecurity among people living with HIV/AIDS [55–58]. These studies found that food insecurity ranged from 16 to 99% with people who are food insecure and also living with HIV/AIDS more likely to experience depression and a lower quality of life [57] and that low income [58] and low education [55] are contributing factors to food insecurity, while ownership of a pressure cooker was found to be protective against food insecurity [56]. Finally, one study explored the experiences of food insecurity among people with tuberculosis [59]. This study found that around 34% of study participants were food insecure, with low income and employment being associated with food insecurity status.

## Discussion

India has seen massive growth and economic change over the past 2 decades; however, this increase in financial wealth has had little impact on food insecurity and population nutrition [60]. While India has increased production and, overall, the availability of food has increased [61], these increases have not yet translated into gains for the general population. Overall, India is seeing increasing income inequality which is having a negative impact on health [62]. As a result of the disconnect between economic growth and positive health outcomes, there has been an increased interest in food insecurity and nutrition in India over the past two decades, resulting in research that seeks to measure food insecurity.

The main finding of this study is the variation in the methods for the assessment of food insecurity prevalence in India and the reliance on cross-sectional studies to elicit food insecurity data. This may be explained by the fact that food security is notoriously difficult to measure. Initial descriptions of food insecurity were conceptualized through the lens of famine [63], meaning that solutions were often confined to domestic agriculture [41]. However, in an increasingly globalized world where countries easily sell and buy goods from each other, it is now important to consider food security in a holistic manner, incorporating the whole definition of food insecurity. By considering the six main dimensions of food security: availability, access, utilization, stability, agency, and sustainability, we can better understand the experiences and drivers of food security. However, as this review has found, few studies measure more than one dimension.

Studies included in this review utilized scales that focused on household food access or availability and were assessed through experience-based scales. Experiential food insecurity scales have been used since the 1990s [64], first used in the USA and later adopted for use in low- and middle-income countries [21, 65]. Experiential measures are based on the notion that food insecurity is associated with a set of knowable and predictable characteristics that can be assessed and quantified [17, 21]. This assumes that households will attempt to mitigate food insecurity through a generalizable or standard pattern of responses [17, 22]. Strategies include reducing expenditure on education expenses [66], selling assets or seeking increased employment [67], and skipping meals or limiting the sizes of meals [68]. Measures of food insecurity that are based on experience seek to capture some of these strategies and actions, and compared to other metrics, such as agriculture production, caloric intake, or anthropometric measures, they enable direct measurement of the prevalence and severity of the extent of household food insecurity, as well as the perception of the quality of their diets [31].

Given the wide variety of measurement tools used, it is difficult to present a comprehensive understanding of food insecurity in India. What is clear is that some households are experiencing food insecurity but are not hungry, while others are both hungry and food insecure. Finding a way to identify and measure at-risk households and intervene to reduce hunger is essential to closing the economic-income gap in India. However, without a measure that can be used consistently across the country that takes into consideration each of the dimensions of food security and the diversity within the Indian population, this will not be possible.

## Limitations

There are some limitations to this review that should also be acknowledged. While every attempt was made to ensure this review was comprehensive, additional articles may have been missed, particularly if articles were written in a language other than English. However, given that this is the first review of its kind, with the inclusion of several databases and broad key terms, the authors are confident that there is little information that is not presented here. The articles presented in this review are largely cross-sectional, and as such, the quality of the studies means that the conclusions drawn by their authors are limited to the study population and are not widely generalizable. The cross-sectional nature of many of the studies limited the potential impact of quality assessment; as such, no quality assessment was conducted. This is a limitation of both this review and the studies included, and in general, a reflection on the rigor with which food security research has been conducted in these settings. Given the variety of approaches taken to measure food insecurity as found in this review, there are challenges in comparing the outcomes of different studies; as such, this review has not sought to present a meta-analysis. If, in the future, there can be some consistency in the use of measurement tools by researchers and agencies, a meta-analysis may be appropriate. The authors do not feel this should invalidate these findings at this time.

## Conclusion

An Indian-specific food security measure needs to be urgently developed and implemented so that food insecurity data can more accurately and consistently be collected and contrasted for the purpose of developing suitable responses to food insecurity. Considering India’s widespread malnutrition and high prevalence of food insecurity, future work should prioritize the development of such a tool in addressing nutrition-related public health in India.

## Supplementary Information

Below is the link to the electronic supplementary material.

Supplementary file1 (DOCX 35 KB)
