# Supplementary material for: Measuring Food Insecurity in India: A Systematic Review of the Current Evidence
Source: Curr Nutr Rep. 2023 Apr 6;12(2):358–67. doi: 10.1007/s13668-023-00470-3 (PMC10264273; doi:10.1007/s13668-023-00470-3)
Supplement: Supplementary file 1 — Supplementary file1 (DOCX 35 KB) [file 13668_2023_470_MOESM1_ESM.docx]

**Table 1: Studies included in review.**

| **Author** | **Year** | Location | Sample size | Design | Primary method | Population | Collect food insecurity | prev food insecurity | Study aim | Food insecurity findings |
| --- | --- | --- | --- | --- | --- | --- | --- | --- | --- | --- |
| Agarwal | 2009 | Delhi | 410 | Cross sectional | Interviews | women | HFSSM - 4 questions | 51% | To explore levels and determinants of experiential household food insecurity and economic access to food | Half of urban slum dwellers were food insecure. Predictors of experienced household food insecurity are directly or indirectly related to low income. |
| Aguayo | 2016 | Maharashtra | 2630 | Cross sectional | survey | Children | CNSM | 43% | To explore stunting in children and to identify policy, program and investment priorities | Over half of households were food secure. |
| Anand | 2019 | Bangalore | 1698 | Cross sectional | Survey | Households | HFIAS | 19% | To explore food security status in Bangalore | The prevalence of food insecurity in this study is in line with other estimates. Food insecurity is related to income and diet diversity. Female headed households are more likely to be food insecure. |
| Anjani | 2012 | India | 100,000 | Longitudinal | survey | Households | NSS | Not reported | To examine food supply, access to food and nutritional security, and policies to address food security | Calorie and protein consumption have declined in rural areas but increased in urban areas over the study period. In poor households, calorie consumption increased marginally. Hunger over the study period declined. |
| Aurino | 2018 | Andhra Pradesh and Telangana | 3000 | Longitudinal | survey | Children | HFIAS | Not reported | To investigate the relation between household food insecurity and child diet | Children identified dietary quality as a critical dimension of their well-being. Children were able to describe and explain the food security challenges of their families and recognize the negative consequences of household food insecurity on their diets, health, and education. |
| Aurino | 2019 | Andhra Pradesh | 1911 | Longitudinal | Secondary survey data analysis from the Young Lives - multi country study | Children | HFIAS | 45% | To assess inequalities in learning and household food security | Overall, household food security at any age predicted lower vocabulary, reading, maths and English scores in early adolescence. Adolescents form households with chronic food insecurity had the lowest scores across all learning outcomes. Identification of necessary approaches needed to target food insecure households to optimise learning and cognitive ability. Wealth was also related to food insecurity as only 34% of households in the lowest wealth quartile were food secure at all three time points while 83% of top wealth quartile households were food secure at all three time points |
| Ayiraveetil | 2020 | South India | 765 | Cross sectional | Survey | Households | HFIAS | 34.10% | To determine level of household food insecurity and its associated factors in patients with pulmonary tuberculosis | Monthly household income less than 3000 rupees, and employment were independently associated with food insecurity. |
| Bagchi | 2018 | West Bengal | 257 | Cross sectional | Survey | Households | Proxy - Per capita consumption expenditure | 15.30% | To assess the incidence, depth, and severity of food inaccessibility | Significant proportion of households with zero operational land and marginal farmers do not have food accessibility. 15.33% of households in the three villages of Birbhum, West Bengal do not had food access. |
| Carpena | 2019 | India | Not reported | Cross sectional | Secondary data analysis | Households | NSS | Not reported | To investigate the impacts of droughts on food expenditure and macronutrient consumption among rural Indian households | Droughts and low rainfall are positively related to food insecurity |
| Chandrasekhar | 2017 | Maharashtra | 2630 | Cross sectional | Secondary data analysis | Children | CNSM | 44.1% | To investigate the relationship between household food security and child's diet diversity and anthropometry | Children from moderately food insecure households are likely to have lower diet diversity. Household food security is related with better infant feeding. |
| Chatterjee | 2012 | Mumbai | 283 | Cross sectional | Interviewer-administered survey | women | HFIAS | 76.3% | To determine the extent of food insecurity in slum households, assess their experiences, and identify subgroups | Food security was associated with lower income. |
| Chinnakali | 2014 | Delhi | 250 | Cross sectional | Semi-structured questionnaire | women | HFIAS | 77.2% | To document the prevalence of food insecurity at the household level and the factors determining its existence in an urban slum population of northern India | Most households had sufficient cereals, but 77.2% of the household were food insecurity. Low monthly per-capita income was a significant independent predictor of food insecurity. The increase in number of working members in household decreased levels of food insecurity in household. |
| Chyne | 2017 | Meghalaya | 510 | Cross sectional | Survey, 24 hour diet recall | women and children | FIES | 67% | To investigate how gender roles and local food resources might contribute to improving the nutritional status of women and children | Despite rich biodiversity in area, undernutrition was high in population. Most of the agricultural produce is going to the market instead of being consumed. The prevalence in children of underweight, stunting, and wasting was high. Nutrient intakes were below recommended levels. |
| Dame | 2011 | Ladakh | 103 | Cross sectional | Household survey, 24 hour diet recall, interviews | Households | Proxy | Not reported | To describe the current food system and understand the use of land | Declining role of agricultural land-use and seasonality leads to periodic food insecurity. |
| Das | 2018 | Karnataka | 200 | Cross sectional | survey | women and children | HFIAS | 33.5% | To determine the prevalence of food insecurity at the household level and to assess the association between food insecurity and malnutrition of under 5 children | Findings indicate an association between food insecurity and age, under-weight, wasting, religion, type of house and education of mother. |
| Dasgupta | 2016 | West Bengal | 173 | Cross sectional | Interviews | Households | proxy Food Security Survey | 49.1% | To identify the prevalence of and factors relating to food security in households of people living with HIV | Over half of participants ate the same food daily and many thoughts food quantity was not adequate. Hindus, members of joint families and those married couples were more likely to be food secure. Coping mechanisms include children being taken out of school or sent to ashrams to earn money; getting loans, or borrowing money from family, friends or neighbours. |
| Dharmaraju | 2018 | Vellore | 150 | Cross sectional | Survey | Households | HFSSM | 57.3% | To re-assess the burden of food insecurity in urban slums | Significant association between household debt and food insecurity. Those households with debts were at higher risk of household food insecurity when compared to household with no debts. |
| Diehl | 2019 | Delhi | 10 | Cross sectional | Interview | Households | HFSSM | Not reported | To understand livelihood and migrant status, household food consumption patterns, and food security | Urban residents who grow their own food may be more food secure because they have greater access and availability of a range of foods. |
| Elsland | 2012 | Aurangabad | 133 | Cross sectional | Survey | Households | HFSSM | 99% | To explore associations between household food security and home gardening, use of soya and pressure cooker ownership in low-income households affected by HIV/AIDS | Home gardening and soya intake were associated with better food security. |
| George | 2000 | Mumbai | 405 | Cross sectional | Survey, 24 hour diet recall | Children | Proxy - ratio of calories consumed vs caloires recommended | 57.40% | To estimate food security among preschool children and to evaluate socio-economic, demographic and anthropometric variables associated with food security | Increase in education levels of mothers increased food security. Consumption of 4 or less meals per day by a child was associated with insecurity. Increasing number of children was associated with increased food insecurity |
| Gopichandran | 2010 | Vellore | 130 | Cross sectional | Survey | Households | HFSSM 18 item | 74.60% | To study the level of food security in a densely populated urban area in southern India. | Food insecurity is higher in this area than in other areas of India. Food insecurity is associated with low income. |
| Heylen | 2015 | Bangalore | 372 | Cross sectional | Interview | Adults | HFIAS | 16% | To understand the experiences of people living with HIV and food insecurity, and to examine the link between food insecurity and mental health | Food insecurity was linked to reduced psychological well-being, especially among men. Food insecurity among people living with HIV/AIDS was lower than other groups |
| Houghton | 2020 | Delhi | 120 | Cross sectional | Survey | Children | HFIAS | 12% | To characterise the feeding and caring practices of disadvantaged urban Indian children 12 to 24 month of age in relation to the WHO recommendations | Stunting, underweight, and wasting was high. Complementary feeding and caregiving practices were sub-optimal |
| Humphries | 2015 | India | 1825 | Cohort Study | Survey, anthropometry | children | HFIAS | 8.70% | To assess associations between food insecurity and stunting and wasting and dietary diversity | Chronically food-insecure households had significantly stunting, although BMI-Zs did not differ by chronic food-insecurity status. |
| Joshi | 2019 | Delhi | 907 | Cross sectional | Survey | Households | HFIAS | 45% | To examine food insecurity in slums | Predictors of food security included household educational level, presence of physically disabled household members, number of household members, coverage of health service needs, electricity needs. Failure to meet employment needs associated with higher food insecurity. |
| Kalyani | 2017 | Odisha | 1161 | Cross sectional | Survey | Households | HFIAS | Not reported | To examine the effects of receiving money on household food security | A higher proportion of women food insecure for each condition and domain in women not receiving Mamata vs receiving Mamata |
| Mahajan | 2015 | India | 100,794 | Cross sectional | Survey | Households | NSS | Not reported | To analyse the effects that food price rises have on calorie and protein intake in India | High income groups are buffered from changes in food prices, but low-income groups who are already malnourished are unable to reduce calorie intake when prices increase. Many low-income households do not have access to the PDS and therefore are at greater risk of malnutrition |
| Maitra | 2015 | Kolkata | 500 | Cross sectional | Survey | Households | HFSSM & Kolkata FSS | 15.4% | To explore the poverty-food security nexus | Poverty is related to food insecurity with low-income households more likely to experience food insecurity. Anti-poverty policies are needed to address food insecurity in India. |
| Maitra | 2017 | Kolkata | 500 | Cross sectional | Survey | Households | HFSSM & Kolkata FSS | 15.4% | To measures experiential food insecurity in low-income urban households of India | Multisectoral interventions are required to address food insecurity, this includes nutritional, education, and poverty reduction interventions. |
| Maitra | 2018 | Kolkata | 500 | Cross sectional | Survey | Households | HFSSM & Kolkata FSS | 15.4% | To examine the association between calorie-based indicators and experiential indicators of food insecurity | Increase in age of household head increases the threat of calorie shortfall possibly because with old age income generation capacity declines. Among the FS household more than 50% are reported to be calorie deprived for different specifications of calorie norm. Household size and age and gender of household head turn out to be strong predictors of both calorie deprivation as well as experiential food insecurity. However, in the former case, household size increases the risk of calorie gap and female head decreases it while, in the previous study, the effects are completely opposite. |
| Manimunda | 2017 | Andaman and Nicobar Isalnds | 1982 | Cross sectional | Survey | Children | HFIAS | 32.60% | To assess prevalence of food insecurity, food and nutrient intake, undernutrition and micronutrient food insecurity in preschool children | Households below poverty line, living in Nicobar region, Christian religion, Nicoabrese tribe, separate kitchen at home, petroleum for cooking, receiving government funding all had protective influence on undernutrition / no sanitary latrine, family income BPL and >6 family members all increased undernutrition. |
| Mastiholi | 2010 | North Karnataka | 700 | Cross sectional | Survey 24 hour diet recalls for half of the sample, blood samples and anthropometry | women | HFIAS | 32% | To assess food insecurity and nutritional status in pre-conception women | Food insecurity was found across all low-income groups. Anaemia was high in women. There was a significant association between food insecurity and anaemia. |
| Maxfield | 2020 | Jaipur | 650 | Cross sectional | Survey | Households | FIES | 66.00% | To assess food and water security in households and assess the relationship between resource insecurity with mental wellbeing | Parents reported more severe overall resource scarcity than children. A larger proportion or parents reported food insecurity and severe water insecurity compared to adolescents. Adults buffer adolescents against resource insecurity, boys more cushioned than girls and mothers more likely to report resource shortages than fathers |
| Mukhopadhyay | 2011 | West Bengal | 15 | Cross sectional | Survey | children | HFSSM | 53.20% | To assess nutritional status and its relation to household food security | Around 2/3 of children were undernourished, increased undernourishment was related to increased food insecurity |
| Mukhopadhyay | 2013 | West Bengal | 245 | Cross sectional | Interview | Children | HFSSM | 80.30% | To assess feeding practices and the association with nutritional status of slum children | Underweight and stunting and wasting were significantly associated with low/very low household food insecurity. |
| Mukhopadhyay | 2010 | West Bengal | 267 | Cross sectional | Survey | Households | HFSSM-Short form | 52.80% | To assess the prevalence of household food insecurity of tribal population | The prevalence of low and very low food security was higher among households having under-5 children. |
| Nishanth Krishna | 2015 | Karnataka | 35 | Cross sectional | Survey | Households | HFSSM | 49% | To assess food security and patterns of nutrient intake, and the relationship between food security with sociodemographic factors | No difference in food insecurity between houses with children, no statically significant results. |
| Nnakwe | 2002 | Coimbatore | 300 | Cross sectional | Interview | Children | Radimer/Cornell | 44% | To measure the prevalence of food insecurity and dietary patterns among households with and without children | Households with children were more likely to be food insecure and at risk of malnutrition |
| Parappurathu | 2019 | Bihar, Jharkhand, Odisha | 480 | Cross sectional | Survey | Households | Proxy - Village Level Studies | Not reported | To measure food consumption patterns, levels of nutrition intake and nutrient intake gap | Diversity of food consumption was low, lacking in nutrients and most calories coming from cereals and vegetables. Significant linkages between nutrient intake and socio-economic and demographic determinants. |
| Patil | 2019 | Pune | 483 | Cross sectional | Survey | Adults | HFIAS | 40% | To understand food security among people living with HIV | The high proportion of people with HIV who are food insecure means that specific interventions should be targeted at this group. |
| Punia | 2019 | Haryana | 300 | Cross sectional | Survey | Households | Proxy Food frequency pattern and household diet diversity survey | Not reported | To assess the food and nutrition security of selected farm households | The majority of families never experienced food scarcity and had enough food to eat for the whole family. Majority of families had medium diet diversity score. Majority of women and children had normal BMI. |
| Rani | 2018 | Varanasi | 418 | Cross sectional | Survey | Teenagers | HFIAS | 47.60% | To investigate the relation between household food insecurity and mental health problems in teenage girls living in urban slums | Teenage girls from food insecure households were more likely to have high levels of anxiety, depression, loss of behavioural control and psychological distress than those living in food secure households |
| Reshmi | 2019 | Bihar, Chhattisgarh, Odisha | 8755 | Cross sectional | Survey, anthropometry | Women | FIES | 77.90% | To assess the context for layering women’s nutrition interventions on a large-scale poverty alleviation program | Access to health services and care to nutritionally “at risk”, access to family planning services, improving dietary diversity, preventing micronutrient deficiencies and anaemia and access to water, sanitation and hygiene services is immense. |
| Sabar | 2016 | Karnataka | 100 | Cross sectional | Survey | Households | NSS | Not reported | To explore the food insecurity level and coping strategies of two particularly vulnerable tribal groups | The availability of food did not guarantee it being accessible. |
| Sajjad | 2014 | Bihar | 959 | Cross sectional | Survey and interviews | Households | Proxy - availability | 74.7% | To measure household level food security | Most families had low food insecurity. The same ranking in terms of large farm, medium and small farm in terms of food security. |
| Sangeetha | 2017 | Uttar Pradesh | 120 | Cross sectional | Survey | Households | proxy - diet diversity | Not reported | To understand dietary habits with special reference to dietary diversity and nutritional status among rural population | Household production enhances food accessibility; hence, promotion of diversified cropping pattern could be a right strategy for enhancing nutritional security in rural areas. Further, this study suggests targeting men as well women for sensitization and awareness programme about importance of food and nutrition to achieve nutritional security of all in particular women |
| Satabdi | 2019 | West Bengal | 240 | Cross sectional | Survey, 24 hour diet recall | Children | HFSSM - 6 questions | 70.90% | To assess if household food security, nutrient adequacy, and childhood nutrition clustered together | Food insecurity was associated with nutritional insecurity |
| Sati | 2017 | Mizoram | 1527 | Cross sectional | Survey | Households | Proxy - food availability | 37.50% | To analyse farming output is related to food security factors | The area faces severe food insecurity and malnutrition but can be transformed with modern agricultural techniques as climate and eco-system are favourable |
| Shubhashis, Lensink and Bhupesh | 2015 | Delhi | 450 | Randomised control trial |  | Households | Proxy - quantity of food consumed | Not reported | To examine the effect of cash transfers rather than the BPL card on nutritional value of household shopping | Unconditional cash transfers do not appear to compromise food security, nor do they induce households to increase wasteful expenses. some evidence that unconditional cash transfers provide opportunities for households to shift to other nutritious, non-cereal options. |
| Sidhu | 2008 | Punjab | 262 | Cross sectional | Unclear | Households | Proxy - calorie requirements and calorie intake of the household | 75% | To study food and nutritional insecurity in a food surplus region | Food insecurity is influenced by income, family size, and urban/rural status |
| Srivastava | 2018 | Rajasthan | 529 | Cross sectional | Survey | Households | HFIAS | 26.00% | To explore the sociodemographic risk factors for household food security and nutrition in desert inhabitants | Households that had income in addition to labour wages were less likely to be food insecure, and larger family size was related to food insecurity |
| Sujoy | 2006 | Gujarat | 1435 | Cross sectional | Survey | Households | proxy - self reported hunger and food availability | 85% | To explore the extent, nature, and dimensions of food insecurity | High degree of food insecurity in vulnerable populations in Gujarat. |
| Tiwari | 2012 | Uttarakhand | 16,080 | Cross sectional | Survey | Population | Proxy - purchasing power | Not reported | To identify the key factors of food insecurity in the Himalayas by analysing the pattern of food production, procurement, and consumption | Households which did not reach nutrition norms were categorized as food insecure. |
